# Use of Neuroendoscopy in Combination Treatment of a Three-Year-Old Patient With Primary Disseminated Medulloblastoma: A Case Report

**DOI:** 10.7759/cureus.68239

**Published:** 2024-08-30

**Authors:** Jalolboy A Ashurov, Gerald Musa, Ella V Kumirova, Sirak N Margaryan, Matvey I Livshitz, Sergei S Ozerov, Gervith Reyes Soto, Manuel De Jesus Encarnacion Ramirez, Gennady E Chmutin

**Affiliations:** 1 Neurological Diseases and Neurosurgery, Peoples' Friendship University of Russia, Moscow, RUS; 2 Neurosurgery, Morozovskaya Children's City Clinical Hospital, Moscow, RUS; 3 Neurosurgery, Livingstone Central Hospital, Livingstone, ZMB; 4 Pediatric Oncology, Morozovskaya Children's City Clinical Hospital, Moscow, RUS; 5 Oncology, Morozovskaya Children's City Clinical Hospital, Moscow, RUS; 6 Neurosurgical Oncology, Mexico National Cancer Institute, Tlalpan, MEX; 7 Neurosurgery, Peoples' Friendship University of Russia, Moscow, RUS

**Keywords:** pediatric oncology, endoscopic biopsy, brain tumor, medulloblastoma, ventriculoperitoneal shunt, etv

## Abstract

A three-year-four-month-old boy with primary disseminated medulloblastoma M3 stage and secondary occlusive hydrocephalus underwent an endoscopic triventriculocisternostomy (ETVC) and tumor biopsy, followed by ventriculoperitoneal shunt (VPS) placement due to ETVC failure. The treatment regimen, which included intensive induction chemotherapy, proton beam therapy (PBT), and maintenance chemotherapy, led to significant clinical improvement and a complete radiological response. Four years post-treatment, the child remains in remission, illustrating the effectiveness of a multimodal approach in managing complex cases of medulloblastoma in pediatric patients.

## Introduction

Over the past decades, significant progress has been made in understanding the biology of medulloblastoma at diagnosis, delineating four molecular subgroups. These subgroups, including WNT, SHH, group 3, and group 4, are now an integral part of the classification of medulloblastoma according to WHO guidelines [1]. Molecular profiling has allowed the identification of second-generation subtypes within groups 3 and 4, which has prognostic value and determines early treatment strategy [2]. Despite these advancements, disease relapse remains the most adverse prognostic factor, occurring in approximately 30% of patients and often leading to fatal outcomes [3,4]. Relapses typically manifest at distant central nervous system (CNS) sites, and while some younger children have experienced long-term remissions with salvage therapy, conventional treatments for older children have shown limited success [5]. The choice of surgical tactics for CSF shunt surgery in patients with secondary hydrocephalus, considering the risk of shunt-associated extraneural metastasis (ENM) in primary tumors of the CNS, is performed on a strict indication [6,7]. We present a clinical case of a child diagnosed with primary disseminated medulloblastoma and secondary hydrocephalus who achieved complete clinical and radiological remission after minimally invasive surgery and chemoradiotherapy.

## Case presentation

A three-year-four-month-old boy presented with vomiting and a fever of up to 38.5°C. Later, he developed ataxia and was admitted to a local hospital. Due to persistent vomiting, a head CT scan was conducted, revealing a tumor obstructing the fourth and left lateral ventricle (LLV), with occlusive hydrocephalus. Consequently, he was urgently transferred to the Morozov Children’s City Clinical Hospital for further investigations and specialized care.

**Figure 1:**
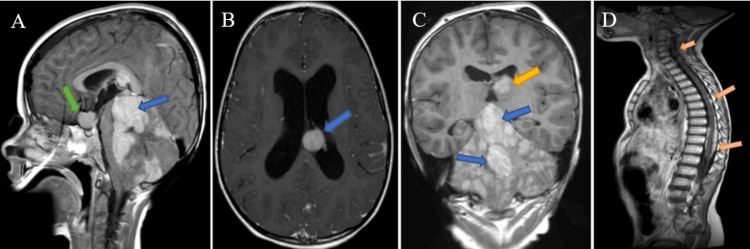
Contrast-enhanced T1 MRI Multifocal lesions of the cerebellum (blue arrow) and suprasellar region (green arrow) (A), LLV (B), left thalamus (yellow arrow), cerebellum and fourth ventricle (blue arrows) (C), and all levels of the spinal cord (D). MRI of the spinal cord was performed after endoscopic biopsy. LLV, left lateral ventricle

During the examination, the child was fully conscious with the rigidity of the occipital muscles, mild tetraparesis (MRC grade 4), ataxia, vertical gaze palsy and lateral strabismus, and bulbar syndrome. Urgent insertion of an external ventricular drain (EVD) was performed to alleviate intracranial pressure (ICP). Subsequent analysis showed negative oncological markers and ruled out endocrine disorders. A contrast-enhanced MRI revealed multiple contrast-enhancing primary disseminated tumors in the cerebellum, fourth and LLV, suprasellar region, and metastasis to the cortex (Figure 1).

Considering the MRI findings and the child's clinical condition, an ETVS was performed, and a biopsy was obtained from the tumor in the LLV in a single-stage procedure.

Surgical procedure

With the patient supine, the ventricle was accessed via the left Kocher's point. The endoscope was inserted into the cavity of the LLV and through the foramen of Monroe into the third ventricle. The anatomy of the third ventricle was altered, with thickening of the floor and pathological tissue in the Sella region. A third ventriculostomy and dilation of the Lilliquist’s membrane with a Fogarty balloon catheter were performed. A neoplasm in the posteromedial aspect of the LLV was visualized and biopsied. Bleeding from the tumor was controlled using monopolar coagulation and NaCl 0.9% irrigation. Considering the altered anatomy of the third ventricle and intraoperative bleeding, an Ommaya reservoir and an EVD were left in situ. The liquoGuard EVD was paused and used to monitor ICP, which remained 11-14 mmHg. The wound was sutured in a standardized manner.

Chest and abdominal CT scans were conducted to exclude extracranial metastasis, yielding no pathology. However, a CNS MRI revealed multiple, primary disseminated tumors of the fourth ventricle, LLV, Sella region, and metastases to the brain and spinal cord. The patient's condition deteriorated rapidly, leading to a stupor, necessitating a transfer to the intensive care unit. Due to the failure of ETVS on day 3, the ICP remained high (11-14 mmHg) and a decision was made to remove the EVD and insert a programmable Medtronic strata 2 ventriculoperitoneal shunt (VPS).

Histological Diagnosis

**Figure 2:**
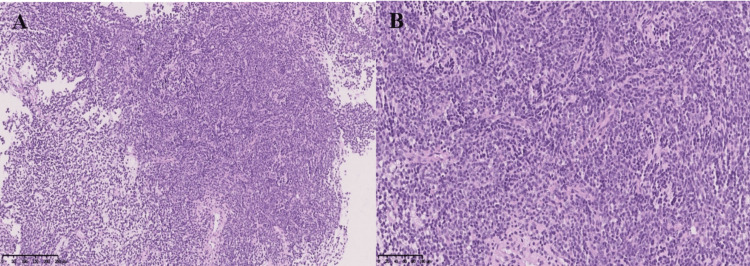
Hematoxylin and Eosin histology stain, X10 (A) and X20 (B) Hypercellular tumor tissue is predominantly medium-sized cells, with a high nuclear-cytoplasmic ratio and areas of apoptosis. The nucleus is hyperchromatic with moderate mitotic activity, rounded and polygonal in shape, the cytoplasm of the cells is practically not visualized with routine staining.

Metastasis of medulloblastoma, NOS, and WHO grade IV malignancy were noted. A molecular genetic study confirmed molecular group 4, MYCN amplification was detected, and TYC was not detected. MGMT was unmethylated, with loss of chromosomes 8, 11, 16q, 17p, and 19 (Figure 2).

Following oncological consultation, a decision was made to perform three blocks of intensive induction chemotherapy according to the HIT MED 2014/2017 protocol (vincristine, cisplatin, cyclophosphamide, etoposide, methotrexate) with intraventricular administration of cytostatic medication, based on the histological examination, the extent of tumor dissemination, and the general condition of the patient. Due to persisting dysphagia and dyspnea, a tracheostomy was performed. Control MRI of the CNS with contrast after two blocks of induction showed a partial response of the main tumor node in the cerebellum and fourth ventricle, as well as a reduction of the size of metastatic foci both in the brain and spinal cord.

**Figure 3:**
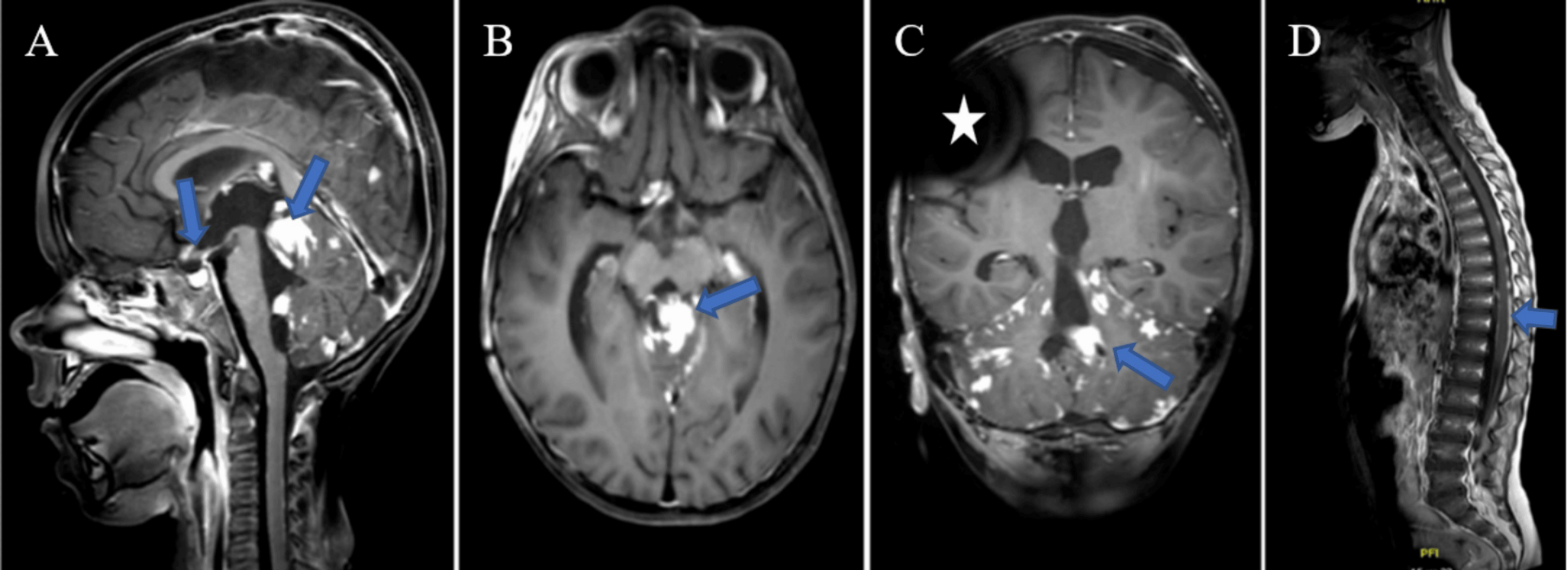
T1 MRI of the CNS three months after the start of IPCT Shows significant tumor shrinkage compared to the first MRI study. In the coronal section (C), a hypointense artifact is seen resulting from the VPS valve (white asterisk). CNS, central nervous system; IPCT, intensive polychemotherapy; VPS, ventriculoperitoneal shunt

Neurologically, the patient improved, which led to the removal of the tracheostomy and the possibility of independent feeding (Figure 3).

After the third block of intensive polychemotherapy (IPCT), MRI of the CNS with contrast showed an increase in the size of metastases subtentorially and in the right thalamus, while the metastases in the spinal cord remained unchanged. Considering clinical data, MRI results, disease progression, tumor histological type, and the age of the child, proton beam therapy (PBT) was performed with sequential treatment of certain areas, without concurrent chemotherapy. Control CNS MRI with contrast after radiotherapy showed a partial response in the form of some reduction of metastases and residual tumors.

Six cycles of maintenance chemotherapy were then given according to the HIT MED 2014/2017 protocol. Post-treatment MRI showed no residual tumor in the posterior cranial fossa, incomplete regression of metastases in the right thalamus, and no lesions throughout the length of the spinal cord. In addition, CSF showed no tumor cells.

**Figure 4:**
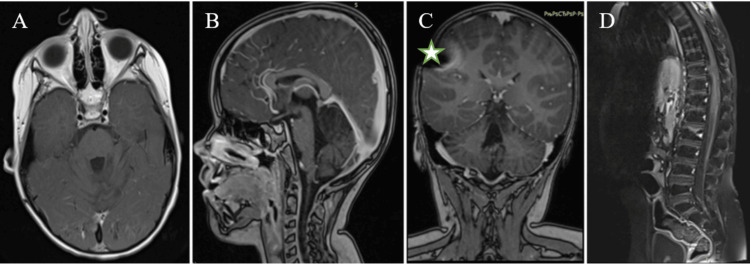
T1 contrast-enhanced MRI of the CNS four years after diagnosis No visible tumor lesions in the CNS. In the coronal section (C), a hypointense artifact is seen resulting from the VPS valve (white asterisk). CNS, central nervous system; VPS, ventriculoperitoneal shunt

Subsequently, six cycles of maintenance mono-chemotherapy with temozolamide were given. Follow-up MRI confirmed complete radiological remission of the tumor. After discharge from the hospital, the child could independently sit and walk albeit with mild ataxia. In the fourth year of outpatient follow-up, a control MRI with contrast showed no tumor in the CNS (Figure 4).

## Discussion

Medulloblastoma, an aggressive pediatric brain tumor, presents challenges due to its propensity for recurrence despite multimodal therapy [8]. In this case, a three-year-four-month-old patient achieved complete remission following chemotherapy and radiotherapy for disseminated medulloblastoma with metastasis to the brain and spinal cord. It is worth noting that the metastasis and associated hydrocephalus may complicate the management of the primary tumor [5]. Single-stage endoscopic triventriculocisternostomy (ETVC) and endoscopic biopsy in the treatment of brain tumors with associated occlusive hydrocephalus remain relevant. The efficacy of ETVC varies with the presence and location of metastases and as technology advances, the molecular groups of tumors are studied [9]. Considering the risk of ENM with shunts in primary CNS tumors with secondary hydrocephalus, neuroendoscopic techniques are preferred [6]. In our case, the ETVC failed, and a VPS with Medtronic Strata 2 programmable valve was inserted. Intraoperative hemorrhage and the presence of liquor metastases reduce the effectiveness of ETVS in patients with tumor hydrocephalus [7].

Chemotherapy, utilizing agents like vincristine, cisplatin, and cyclophosphamide, aims to reduce tumor burden and eradicate micrometastases [10]. However, there is no consensus on the chemotherapy regimen for metastatic disease in children, and its efficacy may be hindered by the blood-brain barrier, necessitating the complementary use of radiotherapy [11,12]. Craniospinal irradiation significantly decreases the risk of local and distant recurrence but is associated with neurocognitive and endocrine sequelae hence not recommended for children under three years of age [13,14]. In this case, the child received chemotherapy with minimal improvement, and a decision was made to switch to PBT and maintenance cycles of chemotherapy. Combination therapy synergistically enhances tumor response and minimizes recurrence risk. By integrating the systemic effects of chemotherapy with targeted radiotherapy, comprehensive tumor control is achieved [9]. Nevertheless, long-term surveillance remains imperative to detect recurrence and manage treatment-related sequelae effectively [1,4]. Four years after discharge, the patient is still in remission but is still being actively followed up with regular MRI screening.

This case underscores the importance of personalized, multidisciplinary approaches in pediatric primary disseminated medulloblastoma management. In cases with secondary complications like hydrocephalus and brain stem symptoms, with multidisciplinary management, these patients can have a good outcome with long-term remission and resolution of neurological deficits. In the presence of endoscopy, a diagnosis can be confirmed without undergoing the highly invasive posterior fossa debulking. To the best of our knowledge, no similar cases have been reported.

## Conclusions

This case illustrates the significant challenges in diagnosing and treating primary disseminated medulloblastoma with secondary occlusive hydrocephalus in pediatric patients. The complexity of the condition required a carefully coordinated, multimodal treatment approach that integrated minimally invasive surgery, intensive chemotherapy, and targeted radiotherapy. The successful management of the case, resulting in sustained remission, underscores the critical importance of a comprehensive treatment strategy that addresses both the primary tumor and its complications. This approach not only led to effective disease control but also improved the overall outcome for the patient, reinforcing the need for a multidisciplinary effort in tackling such aggressive and widespread pediatric brain tumors.
